# Ecological Characteristics of Large-Bodied Sharks in the East Sea of Korea

**DOI:** 10.3390/ani15202974

**Published:** 2025-10-14

**Authors:** Gi Chang Seong, Jeong-Ik Baek, Jong-Ku Gal, Sun-Kil Lee, Jeong-Min Shim, Maeng-Jin Kim

**Affiliations:** East Sea Fisheries Research Institute, National Institute of Fisheries Science, Gangneung 25435, Republic of Korea; sgc0720@naver.com (G.C.S.);

**Keywords:** large-bodied shark, East Sea (Korea), ecological characteristic

## Abstract

**Simple Summary:**

Large sharks are top predators that help maintain balance in marine ecosystems; however, little is known about them in Korean waters. This study provides the first comprehensive overview of large-bodied sharks in the East Sea of Korea. Six species were recorded, with shortfin mako, salmon, and blue sharks most frequently observed during the summer months. Age analysis showed that shortfin makos were mainly juveniles, whereas salmon and blue sharks were generally adults. Overall, diets were predominantly fish, but feeding preferences differed among species. These results indicate that the East Sea serves as an important feeding ground and nursery habitat for some sharks. Overall, this study establishes a baseline for understanding large sharks in the region and helps guide conservation and public safety measures.

**Abstract:**

Large-bodied sharks are key apex predators in marine ecosystems; however, ecological data from Korean waters are limited. From February to November 2024, 44 individuals from six species were collected. Of these, 24 individuals were analyzed for ecological characteristics: shortfin mako (*Isurus oxyrinchus*, *n* = 6), salmon shark (*Lamna ditropis*, *n* = 11), blue shark (*Prionace glauca*, *n* = 6), and great white shark (*Carcharodon carcharias*, *n* = 1). Age and growth parameters were estimated from vertebral band counts using the von Bertalanffy growth model. Diet was assessed via stomach content and DNA metabarcoding, and trophic relationships were examined using stable isotopes. The monthly occurrence peaked in July, with shortfin mako, salmon shark, and blue shark being the most frequently observed species. Estimated ages ranged 8–16, 4–13, and 1–11 years, respectively. The diets were predominantly fish-based, with species-specific prey preferences. Stable isotope data revealed trophic differentiation, suggesting niche partitioning among species. The eastern coastal waters of Korea appear to serve as foraging grounds and potential nursery habitats for large shark species. This is the first comprehensive ecological baseline for these species in Korean waters, which supports future assessments, conservation, and management strategies.

## 1. Introduction

Sharks are apex predators in marine ecosystems that play a role in maintaining ecological balance by regulating the population size and structure of other species [1]. However, most sharks exhibit K-selected life-history traits, such as slow growth, late maturity, and low reproductive rates, which make them highly vulnerable to overfishing and limit their capacity for population recovery. For these reasons, many shark species are listed as threatened on the International Union for Conservation of Nature (IUCN) Red List [2,3]. This further underscores the need for research on the management and conservation of shark resources [4].

Globally, shark populations are declining due to overfishing, bycatch, and habitat destruction [5,6], while rising water temperatures driven by climate change are altering their distribution [7]. In the northwestern Pacific, the shortfin mako shark (*Isurus oxyrinchus*) has shown an overall increasing trend in standardized catch per unit effort (CPUE), but declines have been observed in certain areas, such as the central North Pacific (north of 20° N and east of 180°) since 2006 [8,9]. In the North Pacific, the blue shark (*Prionace glauca*) saw a decline in catch from 2005 to 2009, but a resurgence in numbers has been observed since the 2010s [9,10]. These fluctuations highlight the dynamic nature of shark populations, underscoring the need for ecological research on aspects such as their distribution and habitat utilization to inform effective conservation strategies and mitigate human impacts [11,12].

Located in the northwestern Pacific, the East Sea has recently exhibited marked changes in the occurrence of large-bodied shark species. In 2022, only one accidental shark bycatch was reported, whereas in 2023, the number surged to 15. Notably, a significant portion of the bycatch comprised blue sharks and shortfin mako sharks, both species considered potential threats to human safety, thereby attracting considerable social attention. Over the past several decades (1971–2021), the average annual surface water temperature in the East Sea of Korea has increased by approximately 2 °C. During this period, the catch of cold-water species, such as Alaska pollock (*Gadus chalcogrammus*) and Japanese sandfish (*Arctoscopus japonicus*), has declined, whereas catches of warm-water species, such as amberjack (*Seriola* spp.) and Japanese jack mackerel (*Trachurus japonicus*), have increased. These trends indicate significant changes in the distribution, occurrence patterns, and ecological characteristics of marine organisms [13]. Such environmental changes in the ocean are also thought to influence the occurrence of large-bodied sharks [14], and identifying their ecological role is essential for understanding the ecosystem structure in the region.

Shark research in the Korean Peninsula has been relatively limited, focusing primarily on feeding habits [15], distribution [16], and the appearance of previously unrecorded species [17,18,19]. More recently, an illustrated guide providing taxonomic and ecological information on shark species in Korean coastal waters was published [20]. In the past, sharks were observed only intermittently and were not recognized as important fishery species; thus, ecological studies on their seasonal occurrence, feeding habits, age, and growth remain insufficient. Shark occurrence data provide a critical foundation for understanding migratory patterns under specific spatiotemporal conditions and for establishing safety measures against potentially hazardous species [21,22]. Studies on feeding habits are essential for clarifying predator–prey relationships and serve as vital inputs for ecosystem-based fisheries management [23]. Likewise, studies on age and growth provide key information on population age structure and growth parameters, forming the basis for resource assessments [24].

In this study, we conducted a comprehensive investigation of the monthly appearance patterns, diet, age, and growth of large-bodied shark species found along the East Sea of Korea. This study aims to elucidate the occurrence and ecological roles of large-bodied sharks, provide fundamental data for resource assessment, and contribute to the development of management strategies for shark conservation and improved safety measures for marine recreation activities.

## 2. Materials and Methods

### 2.1. Data Collection and Sampling

**Figure 1:**
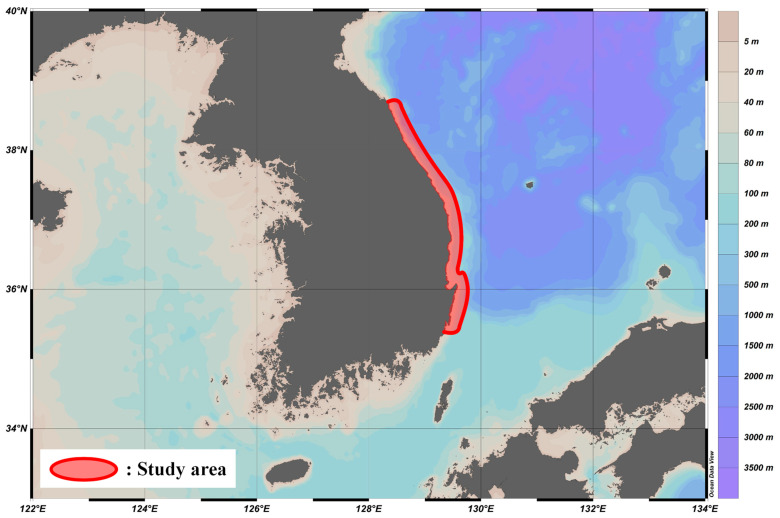
Spatial extent of the study area in the East Sea of Korea, outlined in red.

From February to November 2024, we recorded monthly shark bycatch from set nets, gill nets, and trawl nets, as well as those caught by fishing, in the waters from Goseong, Gangwon Province, to Ulsan (Figure 1). All shark specimens analyzed were obtained from incidental bycatch of commercial fisheries and recreational fishing along the eastern coastal waters of South Korea. Since no animals were deliberately captured or sacrificed, ethical review and approval were not required for this study. In order to examine the ecological traits of each species, the total length and body weight of 24 individuals (six shortfin makos, eleven salmon sharks, six blue sharks, and one great white shark) were measured in the laboratory [20].

### 2.2. Age and Growth Analysis

**Figure 2:**
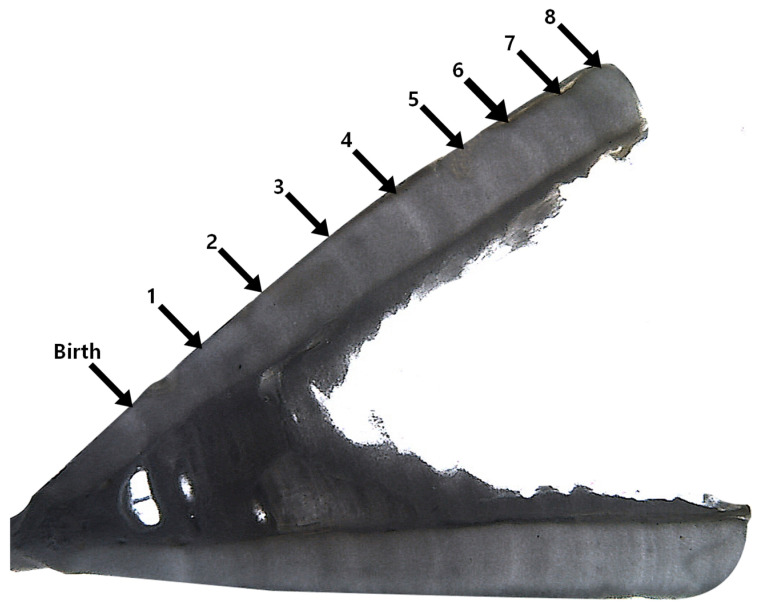
Example image of a vertebral section from a shortfin mako shark estimated to be 8 years old. Arrows indicate the birth mark (Birth) and subsequent growth bands (1–8).

To determine shark age, vertebrae 1–10 were extracted starting from the head. The vertebrae were soaked in commercially available bleach (NaClO) for approximately 24 h to remove soft tissue. After embedding in epoxy resin, 8 μm thick cross-sections passing through the center (focus) of the vertebra were prepared using a low-speed diamond disk cutter. The slices were then polished to enhance the visibility of growth bands, which were observed using a stereomicroscope (Figure 2). Growth bands were counted from the boundary of the translucent zone to the opaque zone, with the first band considered as the birth band [25,26,27,28]. Counts were taken from the focus of the vertebral centrum to the outermost margin. The vertebral and band radii were measured to the nearest 0.01 mm. The vertebral centrum radius (VR) represents the distance from the center to the outermost margin of the centrum, and the band radius (R_n_) is the distance from the center of the centrum to the nth band.

The relative growth equation between the vertebral centrum radius and total length (TL) was estimated [29]. The total length at the time of annulus formation was derived from the mean ring radius, and based on this, the growth equation was estimated using the von Bertalanffy growth model [30].

$$L_{t}=L_{\infty }\times (1-e^{-k\left(t-t_{0}\right)})$$

Here, *L_t_* refers to the TL at age *t*, *L_∞_* is the theoretical maximum TL, *k* represents the growth coefficient, and *t*_0_ denotes the theoretical age when TL is 0. The parameters of the growth equation were first estimated using the Walford plot method [31] to obtain the estimates (*L_∞_*, *k*, *t*_0_), and then further refined using nonlinear regression analysis to minimize the error between the data and the growth curve. Ninety-five percent confidence bands of the estimated growth curves and 95% confidence intervals of the growth parameters were obtained by bootstrapping with 2000 replicates. The statistical analysis was carried out using R software (version 4.4.3) [32].

### 2.3. Morphological Diet Analysis

To determine the main food sources, stomach contents were analyzed after dissection. The identified prey organisms were classified to the lowest possible taxonomic level [33,34], counted, and weighed. The results of the prey analysis were quantitatively expressed as frequency of occurrence (%*F*), numerical proportion (%*N*), and weight proportion (%*W*), calculated using the following formulas:

$$\%F=\frac{A_{i}}{N}\times 100$$

$$\%N=\frac{N_{i}}{N_{Total}}\times 100$$

$$\%W=\frac{W_{i}}{W_{Total}}\times 100$$

where *A_i_* is the number of fish preying on species *i*, *N* is the total number of fish examined (excluding individuals with empty stomachs), *N_i_* (*W_i_*) is the number (wet weight) of prey species *i*, and *N_total_* (*W_total_*) is the total number (wet weight) of prey. The index of relative importance (*IRI*) of the ingested prey items was calculated using the formula [35] and expressed as follows:

$$IRI=\left(\%N+\%W\right)\times \%F$$

The relative importance index ratio (%*IRI*) is the relative importance index converted into a percentage, as shown in the following formula:

$$\%IRI=\frac{{IRI}_{i}}{\sum_{j=1}^{n}IRI}\times 100$$

### 2.4. Metabarcoding Analysis

To conduct molecular biological analysis on the above contents using metabarcoding, the weight of the sample was measured, and six times its weight of lysis buffer was added, followed by primary homogenization using a blender. Complete homogenization was achieved using a homogenizer (FastPrep-24 5G, MP Biomedicals, Irvine, CA, USA). Total genomic DNA was extracted according to the manufacturer’s protocol for the DNeasy Blood & Tissue Kit (Qiagen, Hilden, Germany).

To analyze the relative proportions of the entire taxonomic group comprising shark food sources, the nuclear 18S ribosomal RNA gene was identified. Subsequently, a multi-marker metabarcoding analysis was conducted using cytochrome c oxidase subunit I (COI) and 12S rRNA markers to determine the food sources of the sharks.

Library construction was performed using primer sets with overhang adapter sequences (Illumina, San Diego, CA, USA) added to each 18S gene marker. Library preparation followed previously described methods [36,37]. After conducting the first round of PCR for each gene marker, electrophoresis was used to confirm the expected band size (approximately 200–300 bp). PCR products were then pooled by shark species and purified using the AccuPrep PCR/Gel DNA Purification Kit (Bioneer, Daejeon, Republic of Korea) following the manufacturer’s protocol. Purified first-round PCR products were subsequently used to construct the final NGS library following the Nextera XT DNA Library Prep Reference Guide protocol, with Nextera Index Kits (Illumina, San Diego, CA, USA). The quality and quantity of the prepared library were assessed using a Quantus Fluorometer and ONE dsDNA kit (Promega, Madison, WI, USA), and sequencing analysis was performed using the MiSeq Reagent Kit v3 (600-cycle; Illumina, San Diego, CA, USA).

Large-scale nucleotide sequence data from NGS results were processed using the CLC Genomic Workbench (CLC Bio, Cambridge, MA, USA) to remove adapters and index sequences and to filter out low-quality reads (QV < 30). Amplicon sequence variants (ASVs) were generated using the DADA2 package [38] to derive accurate nucleotide sequences. Taxonomic assignment of generated ASVs was performed using the NCBI reference database and the Basic Local Alignment Search Tool. Based on sequence similarity, ASVs were classified as species (≥99%), genus (90–98%), or unknown (<90%).

Dietary overlap among shark species was quantified using Schoener’s index (*C_x__y_*), which is widely used in trophic ecology to evaluate the degree of dietary similarity between consumers. Schoener’s index was calculated using the formula [39] and expressed as follows:

$$C_{xy}=1-0.5(\sum_{i=1}^{n}\left|P_{xi}-P_{yi}\right|)$$

where *P_xi_* and *P_yi_* represent the proportions of prey item *i* in the diets of species *x* and *y*. The index values range from 0 (no overlap) to 1 (complete overlap), with values above 0.6 generally considered to indicate biologically significant dietary overlap. Prey composition was derived from metabarcoding analyses, based on the relative proportions of prey organisms identified through COI and 12S rRNA markers.

### 2.5. Stable Isotope Analysis

To determine the isotopic niche of the shark species, muscle tissue was collected from individual specimens and stored frozen until analysis. The tissue was then freeze-dried and homogenized using a mortar and pestle. Approximately 1 mg of each sample was placed in a tin capsule and sealed.

Carbon and nitrogen stable isotope ratios were determined using an elemental analyzer coupled with an isotope ratio mass spectrometer. International isotopic references (IAEA-CH-6 and N-1) were used as running standards and to ensure analytic precision. Results were expressed using the δ notation for carbon and nitrogen isotopes as follows:

$$\delta X\left(‰\right)=\left[\left(\frac{R_{sample}}{R_{standard}}\right)-1\right]\times 10^{3}$$

Here, *R_sample_* represents the ratio of ^13^C/^12^C or ^15^N/^14^N in the sample, and *R_standard_* denotes the ratio in the Vienna Pee Dee Belemnite and atmosphere, respectively.

To evaluate the trophic niche, we applied the Stable Isotope Bayesian Ellipses in R (SIBER) package [40]. Comparisons of isotopic niche size and ellipse areas were performed using the total area (TA) and small sample size-corrected standard ellipse area (SEAc), a quantitative proxy for the trophic diversity of sharks based on the spread and extent of isotopic data points [41]. Two indices were estimated using a conservative estimate of the maximum potential overlap among sharks and considered the uncertainties and biases that occur due to small or differing sample sizes [40].

## 3. Results

### 3.1. Monthly Occurrence

**Figure 3:**
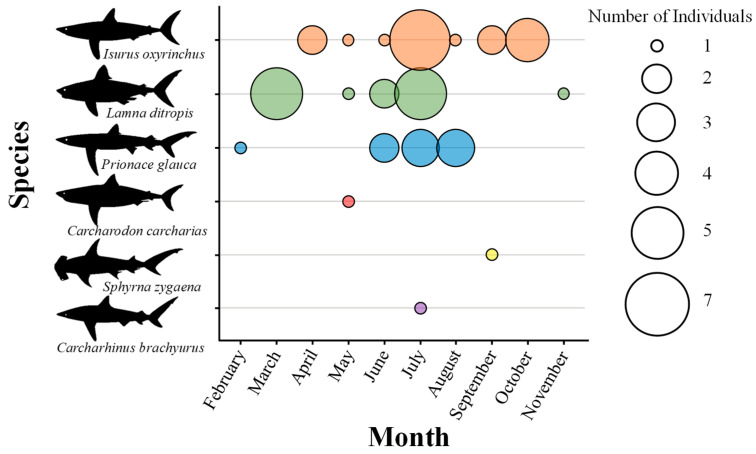
Monthly changes in the number of shark individuals recorded in the East Sea of Korea, February–November 2024. Colored circles represent each shark species: orange, *Isurus oxyrinchus*; green, *Lamna ditropis*; blue, *Prionace glauca*; red, *Carcharodon carcharias*; yellow, *Sphyrna zygaena*; purple, *Carcharhinus brachyurus*, and circle size indicates the number of individuals observed.

A total of 44 individuals representing six species of large-bodied sharks were identified (Table A1), namely, the shortfin mako (*Isurus oxyrinchus*), salmon shark (*Lamna ditropis*), blue shark (*Prionace glauca*), smooth hammerhead (*Sphyrna zygaena*), great white shark (*Carcharodon carcharias*), and copper shark (*Carcharhinus brachyurus*). Bycatch in set nets accounted for the highest proportion at 79.1%, followed by gillnets at 14.0%, fishing lines at 4.7%, and trawl nets at 2.3%. Analysis of monthly changes in shark bycatch from February to November (Figure 3) showed that the largest number of individuals (16) was recorded in July, while the lowest counts (1) were recorded in both February and November. For the shortfin mako, seven and four individuals were caught in July and October, respectively; for salmon sharks, five individuals were caught in March and July; and for blue sharks, three individuals were found in July and August. Notably, all three dominant species recorded in this study exhibited peak bycatch in July.

### 3.2. Age and Growth

**Figure 4:**
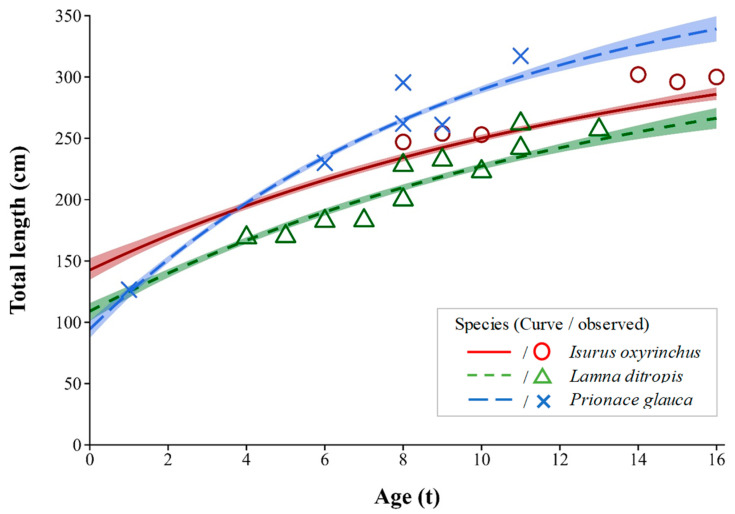
Length-at-age relationship of shark species occurring in the East Sea of Korea. Lines indicate von Bertalanffy growth curves with 95% confidence intervals (shaded areas); symbols represent observed length-at-age data.

Age estimation based on the growth rings observed in the vertebrae (Figure 4) revealed that shortfin makos (*n* = 6) ranged from 8 to 16 years old, salmon sharks (*n* = 11) from 4 to 13 years old, and blue sharks (*n* = 6) from 1 to 11 years old. Based on the relationship equations between vertebral ring counts and total length for each species, the average total length by age was as follows: shortfin mako, TL_8_ = 247.0 cm and TL_16_ = 300.0 cm; salmon shark, TL_4_ = 247.0 cm and TL_13_ = 300.0 cm; and blue shark, TL_1_ = 126.6 cm and TL_11_ = 317.2 cm. In addition, using the average total length at each age, the estimated VBF growth equations derived by non-linear regression were as follows (Table 1): shortfin mako, $L_{t}=352.6\left(1-e^{-0.072\left(t+7.23\right)}\right)$; salmon shark, $L_{t}=338.3\left(1-e^{-0.073\left(t+5.37\right)}\right)$; blue shark, $L_{t}=395.8\left(1-e^{-0.104\left(t+2.61\right)}\right)$.

### 3.3. Diet Analysis

**Figure 5:**
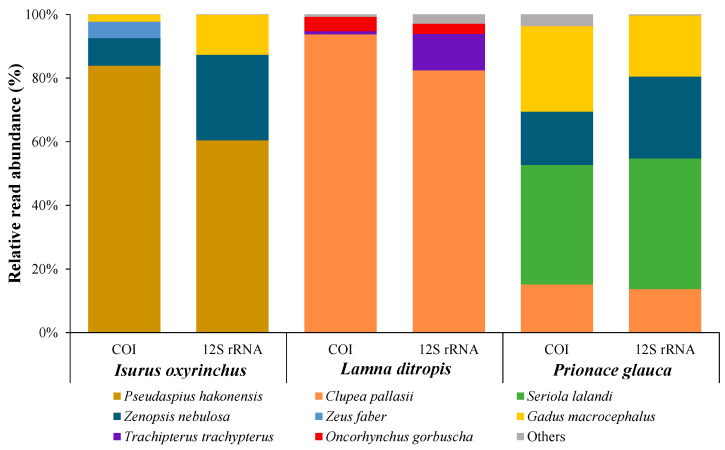
Prey composition of three dominant shark species identified by COI and 12S rRNA metabarcoding.

Analysis of stomach contents of the three dominant species (shortfin mako, salmon shark, and blue shark) in this study (Table 2) revealed that fish were the primary prey, with relative importance index values of 99.6%, 73.7%, and 90.1%, respectively. The short-fin mako shark primarily consumed dories (Zeidae), the salmon shark primarily ate slender ribbonfish (*Trachipterus ishikawae*) and Pacific herring (*Clupea pallasii*), and the blue shark primarily fed on Pacific cod (*Gadus macrocephalus*). Prey items identified using the COI and 12S rRNA markers included eight species for the shortfin mako, seven species for the salmon shark, and nine species for the blue shark, totaling 19 prey species (Figure 5), predominantly fish. For the shortfin mako, the big-scaled redfin (*Pseudaspius hakonensis*) was the dominant prey, accounting for 83.9% and 58.8% in the COI and 12S rRNA markers, respectively. For the salmon shark, Pacific herring was the most dominant, corresponding to 94.4% and 81.7% of the COI and 12S rRNA markers, and for the blue shark, yellowtail amberjack (*Seriola lalandi*) was the most dominant at 38.7% and 41.1%, respectively. Schoener’s index revealed a very low dietary overlap among the three shark species examined (Table 3). The overlap between shortfin mako and salmon shark was almost negligible (C = 0.0001), while overlap between shortfin mako and blue shark was 0.381. The overlap between the salmon and blue sharks was similarly low (0.137). These results indicate that the three shark species exhibit clear dietary segregation, reflecting distinct trophic niches and minimal competition for prey resources in the East Sea.

**Figure 6:**
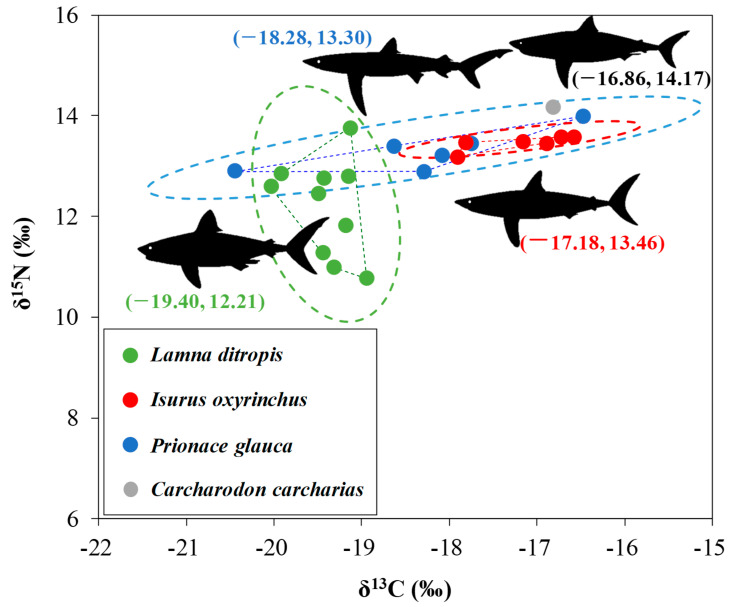
Carbon (δ^13^C, ‰) and nitrogen (δ^15^N, ‰) stable isotope ratios of four large-bodied shark species collected from the East Sea of Korea. Individual points denote sampled specimens, polygons indicate the total areas (TA), and dashed ellipses (dashed circles) indicate the isotopic niches (SEAc) estimated by the Stable Isotope Bayesian Ellipses in R (SIBER). Values in parentheses represent the mean δ^13^C and δ^15^N values for each species.

The average isotopic values of carbon and nitrogen were −17.18‰ and 13.46‰ for the shortfin mako, −19.40‰ and 12.21‰ for the salmon shark, −18.28‰ and 13.30‰ for the blue shark, and −16.86‰ and 14.17‰ for the great white shark, respectively (Figure 6). Shortfin mako exhibited the narrowest isotopic niche (TA = 0.21, SEAc = 0.21), while blue shark showed intermediate values (TA = 1.21, SEAc = 1.24) than other sharks. The salmon shark displayed the largest TA (1.73) but a relatively smaller SEAc (1.12), suggesting a greater isotopic spread but a more constrained core niche. The isotopic niches showed moderate overlap between mako and blue sharks (16.8%) and between blue and salmon sharks (15.9%), whereas the overlap between mako and salmon sharks was negligible (<0.01%). The stable isotope data further suggest that salmon sharks mainly prefer cold-water prey, in contrast to the warm-water foraging habits of shortfin makos and blue sharks, with the latter exhibiting a relatively broader isotopic niche.

## 4. Discussion

### 4.1. Occurrence Patterns

In this study, the observed abundance of large-bodied sharks in the East Sea differed markedly from previous surveys. Between 2011 and 2016, a total of 17 shortfin makos, 14 salmon sharks, 10 blue sharks, 5 great white sharks, and 4 hammerhead sharks were reported in the East Sea of Korea [14]. In contrast, the present study recorded 44 individuals within less than one year, indicating a relatively higher number of specimens on a per-year basis. Notably, the high occurrence of shortfin makos, which inhabit temperate and tropical waters, suggests a possible shift in their migration patterns due to changes in the marine environment [9,42]. The distribution and migration paths of these sharks are influenced by environmental and ecological factors, such as water temperature, salinity, and prey availability, which often act in combination [43,44,45]. Considering the close correlation between food distribution and shark movements [46], the increased abundance of sharks in the East Sea and their concentration in summer likely reflect favorable environmental conditions and seasonal increases in prey availability [47,48,49]. However, because this study is based on short-term data, continued monitoring is necessary to assess long-term trends in shark occurrence. Additionally, further research on environmental factors, such as water temperature, food resources, and ocean currents, is required to better interpret these trends. Such comprehensive analyses will enhance the accuracy of models predicting changes in shark distribution.

### 4.2. Age and Growth Characteristics

Age and growth data are essential for building population models and provide a foundation for establishing sustainable fishery management and conservation strategies [50]. Common structures used to estimate the age of fish include otoliths, scales, and vertebrae. For shortfin mako, salmon, and blue sharks, a single growth ring forms annually in the vertebrae, making them suitable for age determination [27,51,52]. In this study, shortfin makos exhibited relatively slow growth rates than salmon and blue sharks, consistent with previous research [27,53,54]. In the North Pacific, the age at 50% maturity differs among shark species: female salmon sharks mature at 6–9 years, males at 3–5 years [27]; female shortfin makos at 17.2 years and males at 5.2 years [55]; and female blue sharks at 3.7 years and males at 4.2 years [56,57]. Based on these results, most shortfin makos observed in this study are presumed to be juveniles, and their slow growth and late maturation make them particularly vulnerable to overfishing. In contrast, most salmon and blue sharks are presumed to be adults. Given that only one mature gonad was found in a blue shark in June, the East Sea may serve as an important nursery and feeding ground for these three species and could potentially function as a spawning ground. However, the relatively small sample sizes analyzed for each species (≤11 individuals) and the short temporal coverage of this study (less than one year) limit the ability to generalize the population age structures, reproductive status, and habitat use. Therefore, while the findings suggest that the East Sea may play a role in the life cycle of these sharks, the precise ecological functions of this region remain uncertain. To better understand their habitat usage patterns, migration routes, and reproductive ecology, further long-term monitoring and large-scale sampling will be essential.

### 4.3. Feeding Ecology

Fish feeding habits are examined to identify key prey species for a given target species and provide insight into ecosystem energy flow, including predator–prey interactions and interspecific competition [58]. Morphological analysis of stomach contents is a commonly used method to quantitatively assess short-term feeding patterns. However, this method is often limited by the inability to identify prey to the species level due to varying degrees of digestion. To overcome these limitations, molecular techniques such as DNA metabarcoding have recently been widely adopted, allowing more accurate identification of prey species [59]. In this study, the diets of the shortfin mako, salmon shark, and blue shark centered on fish, indicating that these species occupy high trophic levels within the ecosystem. Pacific herring form large schools and migrate to shallow coastal waters for spawning during spring (March–May) [33], and both Pacific herring and salmon sharks prefer cold water temperatures. This overlap in timing and distribution likely explains the dominance of herring in the stomach contents of the salmon sharks. Additionally, big-scaled redfin are known to mostly inhabit the sea but migrate to rivers for spawning from March to June, making them a likely primary prey species for shortfin makos during this period. In contrast, blue sharks consumed a wider variety of prey, with yellowtail forming schools in the East Sea during summer serving as a key food resource. In conclusion, while these three shark species can compete as top predators within the ecosystem, the salmon shark is spatially separated from the others because of its physiological preference for lower temperatures, and the broad diet of the blue shark provides a competitive advantage in interspecific food competition, enabling the coexistence and shared habitat use among all three species. The prey composition observed in this study may provide insight into prey availability on a limited spatiotemporal scale. Moreover, shifts in prey communities due to marine environmental shifts can directly impact shark distribution and their ecological roles as predators [60,61]. Therefore, information on shark feeding habits can serve as a sensitive indicator of ecosystem changes [62]. Accordingly, while our results provide a valuable short-term baseline, further long-term and large-scale studies are needed to better understand future ecosystem function and structure under climate change in the region.

## 5. Conclusions

This study is the first to comprehensively analyze the monthly fluctuations in the occurrence, growth, and diet of sharks in the East Sea of Korea. The feeding ecology of sharks was examined using three analytical methods—stomach content analysis, DNA metabarcoding, and stable isotope analysis—applied in parallel, enabling the identification of ecological niches and dietary partitioning among shortfin mako, salmon, and blue sharks. These species occupy high trophic levels, exerting a significant influence on the population dynamics of lower trophic levels and playing a crucial role in maintaining the balance of the East Sea ecosystem. The vertebral analysis in this study contributed to the understanding of growth parameters, and occurrence records suggest that the East Sea may provisionally serve as a foraging and nursery ground.

The implications of these findings for conservation are considerable. The shark species identified in this study exhibit a range of conservation statuses on the IUCN Red List, from Least Concern to Endangered, underscoring the necessity of continued monitoring and management. However, their life-history traits, such as slow growth, delayed maturity, and low reproductive rates, render them particularly susceptible to overexploitation. Therefore, it is crucial to implement management strategies aimed at reducing human-induced mortality, such as enhancing fishing practices to minimize shark bycatch [63]. In terms of human safety, shark-related incidents in Korean coastal waters have historically been rare, with six cases reported between 1959 and 1996 (one involving a swimmer and five involving fishing activities). Although no such incidents have been documented in the East Sea, reports of large shark bycatch have gradually increased in recent years. In July 2025, a blue shark measuring approximately 2 m in total length was recorded near a bathing beach. Because shark occurrence peaks in summer, coinciding with the beach season, heightened vigilance is required to prevent shark attacks. In addition, with an increasing number of large shark sightings, safety measures should be introduced, including advanced tracking technologies and alert systems for shark monitoring initiatives [64]. Although this study expands the ecological baseline, its short duration and limited sample size necessitate caution. Future studies should extend monitoring, expand sample collections, and integrate remote tracking with genetic analyses to ascertain the distribution and migration routes, population connectivity, and potential species differentiation [65]. Such integrated research will support evidence-based management and conservation while informing strategies to mitigate social and safety concerns associated with large sharks in the East Sea.

## Figures and Tables

**Table 1:** Estimates of von Bertalanffy growth parameters for three shark species occurring in the East Sea of Korea.

Species	*n*	Estimates of Parameters			95% Confidence Intervals		
		*L_∞_*	*K*	*t*_0_	*L_∞_*	*K*	*t*_0_
*Isurus oxyrinchus*	6	352.6	0.072	−7.23	320.3–432.6	0.043–0.098	−10.07–−5.57
*Lamna ditropis*	11	338.3	0.073	−5.37	295.5–431.2	0.043–0.103	−7.07–−4.07
*Prionace glauca*	6	395.8	0.104	−2.61	365.5–439.2	0.083–0.127	−3.16–−2.15

**Table 2:** Composition of stomach contents of three large shark species from the East Sea of Korea by frequency of occurrence (%*F*), number (%*N*), weight (%*W*), and index of relative importance (%*IRI*).

Species		*Isurus oxyrinchus*					*Lamna ditropis*					*Prionace glauca*				
Prey organism		%*F*	%*N*	%*W*	*IRI*	%*IRI*	%*F*	%*N*	%*W*	*IRI*	%*IRI*	%*F*	%*N*	%*W*	*IRI*	%*IRI*
Pisces		100.0	96.0	99.99	19,599.4	99.6	80.0	31.6	95.8	10,190.4	73.7	83.3	67.6	99.8	13,954.9	90.1
	*Aptocyclus ventricosus*						10.0	1.3	9.1							
	*Clupea pallasii*						20.0	10.5	8.6			16.7	23.5	41.9		
	*Gadus macrocephalus*											50.0	14.7	21.4		
	*Gymnocanthus herzensteini*											16.7	5.9	7.1		
	*Hippoglossoides pinetorum*	20.0	4.0	3.8												
	Monacanthidae	20.0	4.0	0.9												
	Pleuronectidae											16.7	11.8	2.9		
	Salmonidae	20.0	8.0	34.4												
	*Scomber japonicus*						10.0	1.3	1.2							
	*Seriola quinqueradiata*											16.7	5.9	10.3		
	*Trachipterus ishikawae*						10.0	1.3	53.7							
	Zeidae	20.0	4.0	40.3								16.7	2.9	16.1		
	Unidentified Pisces	60.0	76.0	20.6			60.0	17.1	23.2			16.7	2.9	0.2		
Cephalopoda		20.0	4.0	+	80.1	0.4	50.0	68.4	4.2	3631.0	26.3	50.0	29.4	0.1	1477.4	9.5
	*Todarodes pacificus*						10.0	1.3	2.3							
	*Watasenia scintillans*											16.7	2.9	0.1		
	Unidentified Cephalopoda	20.0	4.0	+			50.0	67.1	1.9			50.0	26.5	+		
Anomura												16.7	2.9	0.1	49.9	0.3
Total			100.0	100.0	19,679.5	100.0		100.0	100.0	13,821.4	100.0		100.0	100.0	15,482.2	100.0

+: Less than 0.1%.

**Table 3:** Schoener’s index of dietary overlap (COI and 12S rRNA) among three large shark species from the East Sea of Korea.

Comparison	COI	12S rRNA
*Isurus oxyrinchus* vs. *Lamna ditropis*	0.0000	0.0001
*Isurus oxyrinchus* vs. *Prionace glauca*	0.1094	0.3808
*Lamna ditropis* vs. *Prionace glauca*	0.1565	0.1369

## Data Availability

The data presented in this study are available from the corresponding author, upon reasonable request. The data are not publicly available because of institutional restrictions.

## Appendix A

**Table A1:** Records of shark individuals in the East Sea of Korea, February–November 2024. Species are abbreviated as SM, shortfin mako (*Isurus oxyrinchus*); SS, salmon shark (*Lamna ditropis*); BS, blue shark (*Prionace glauca*); SH, smooth hammerhead (*Sphyrna zygaena*); WS, great white shark (*Carcharodon carcharias*); CS, copper shark (*Carcharhinus brachyurus*). M, male; F, female. NA indicates data not available.

No.	Date	Gear	Species	Location	Total Length (cm)	Body Weight (kg)	Sex
1	7 February 2024	Set net	BS	Uljin	126.6	7.0	M
2	21 March 2024	Set net	SS	Uljin	200.0	96.6	F
3	24 March 2024	NA	SS	Goseong	NA	NA	NA
4	25 March 2024	Set net	SS	Goseong	170.1	43.4	M
5	25 March 2024	Set net	SS	Goseong	182.5	62.5	M
6	25 March 2024	Set net	SS	Goseong	169.0	43.8	M
7	22 April 2024	Set net	SM	Sokcho	295.0	160.0	NA
8	26 April 2024	Set net	SM	Donghae	247.0	92.0	F
9	7 May 2024	Set net	SM	Uljin	253.0	113.7	F
10	25 May 2024	Set net	WS	Uljin	205.0	76.2	F
11	31 May 2024	Set net	SS	Sokcho	243.0	150.0	F
12	4 June 2024	Gill net	BS	Ulsan	NA	NA	NA
13	11 June 2024	Set net	BS	Gangneung	295.5	122.3	F
14	24 June 2024	Gill net	SS	Samcheok	257.0	186.6	F
15	26 June 2024	Set net	SS	Uljin	262.0	168.4	F
16	28 June 2024	Trawl	SM	Uljin	NA	NA	NA
17	2 July 2024	Set net	BS	Uljin	201.0	35.4	M
18	8 July 2024	Set net	SS	Uljin	223.0	124.3	F
19	10 July 2024	Set net	BS	Gangneung	261.0	96.0	M
20	14 July 2024	Set net	SM	Goseong	100.0	7.5	NA
21	17 July 2024	Gill net	SS	Uljin	183.0	87.3	M
22	19 July 2024	Set net	SM	Samcheok	126.0	NA	NA
23	19 July 2024	Set net	SM	Samcheok	120.0	NA	NA
24	19 July 2024	Set net	SM	Samcheok	112.0	NA	NA
25	20 July 2024	Set net	SS	Donghae	NA	NA	NA
26	20 July 2024	Set net	BS	Donghae	NA	NA	NA
27	22 July 2024	Set net	SM	Uljin	254.0	70.4	F
28	22 July 2024	Set net	SM	Samcheok	121.0	NA	NA
29	25 July 2024	Set net	CS	Uljin	77.9	2.4	F
30	25 July 2024	Set net	SM	Yeongdeok	NA	NA	NA
31	26 July 2024	Set net	SS	Yeongdeok	232.4	112.2	M
32	30 July 2024	Set net	SS	Gangneung	228.0	142.4	F
33	5 August 2024	Set net	BS	Uljin	262.0	78.4	M
34	7 August 2024	Set net	BS	Uljin	317.2	128.9	M
35	17 August 2024	Fishing	SM	Samcheok	320.0	NA	NA
36	27 August 2024	Gill net	BS	Uljin	230.0	55.8	M
37	10 September 2024	Set net	SH	Uljin	179.0	21.8	F
38	11 September 2024	Set net	SM	Uljin	100.0	9.0	F
39	13 September 2024	Fishing	SM	Samcheok	NA	NA	NA
40	14 October 2024	Set net	SM	Uljin	302.0	226.9	F
41	16 October 2024	Set net	SM	Uljin	296.0	184.4	F
42	18 October 2024	Set net	SM	Uljin	300.0	229.5	F
43	31 October 2024	Gill net	SM	Uljin	NA	NA	NA
44	12 November 2024	Gill net	SS	Donghae	242.0	114.5	M
